# The impact of sleep duration, sleep debt and insomnia symptoms on infection risk: a longitudinal cohort study with 1-year follow-up

**DOI:** 10.1093/fampra/cmag018

**Published:** 2026-04-20

**Authors:** Bjørn Bjorvatn, Siri Waage, Ragnhild S Lundetræ, Leo Larsen, Nadia Pristaj, Magnus Fossum, Ingrid K Rebnord, Knut Erik Emberland

**Affiliations:** Department of Global Public Health and Primary Care, University of Bergen, N-5009 Bergen, Norway; Norwegian Competence Center for Sleep Disorders, Haukeland University Hospital, N-5021 Bergen, Norway; Department of Global Public Health and Primary Care, University of Bergen, N-5009 Bergen, Norway; Norwegian Competence Center for Sleep Disorders, Haukeland University Hospital, N-5021 Bergen, Norway; Department of Global Public Health and Primary Care, University of Bergen, N-5009 Bergen, Norway; Department of Global Public Health and Primary Care, University of Bergen, N-5009 Bergen, Norway; Department of Global Public Health and Primary Care, University of Bergen, N-5009 Bergen, Norway; Department of Global Public Health and Primary Care, University of Bergen, N-5009 Bergen, Norway; Department of Global Public Health and Primary Care, University of Bergen, N-5009 Bergen, Norway; Department of Global Public Health and Primary Care, University of Bergen, N-5009 Bergen, Norway

**Keywords:** insufficient sleep, insomnia severity, infections, follow-up study, PraksisNett, practice-based research network

## Abstract

**Background:**

To study whether sleep duration, sleep debt (difference between sleep need and duration), and insomnia severity at baseline would predict increased prevalence of infections 1 year later.

**Methods:**

In total, 861 participants recruited from the Norwegian Primary Care Research Network completed a baseline survey about sleep and were invited for follow-up 1 year later. The response rate at follow-up was 60.5%, rendering 521 patients eligible for analyses. At baseline the participants completed questions about sleep habits and insomnia symptoms (insomnia severity index), and at follow-up they responded to whether they had experienced various infections during the last 3 months and whether they experience more infections than peers.

**Results:**

Participants with normal sleep duration, no sleep debt and no insomnia at baseline reported the lowest prevalences of infections at 1-year follow-up. In the adjusted analyses, long sleep duration (>9 h, present only in 11 participants) increased the odds of influenza-like illness, gastrointestinal infection, and experiencing more infections than peers by about four times, whereas short sleep duration (<6 h) doubled the odds of skin infection. Compared to no sleep debt, ≤2 h sleep debt doubled the odds of skin infection and >2 h sleep debt tripled the odds of experiencing more infections than peers. Insomnia doubled the odds of any type of infection and influenza-like illness, and more than tripled the odds of gastrointestinal infection and experiencing more infections than peers.

**Conclusions:**

This longitudinal study supports the notion that people who have insufficient sleep are at increased risk of infections.

Key messages

- Few longitudinal studies investigate a possible link between sleep and infections
- We assessed sleep at baseline and prevalence of infections at 1-year follow-up
- Short and long sleep durations increased risk of infections at follow-up
- Sleep debt (need minus duration) increased risk of infections at follow-up
- Insomnia symptoms increased risk of infections at follow-up
- We conclude that insufficient sleep increases infection risk at 1-year follow-up

## Introduction

Sleep is vital to health and well-being. Insufficient sleep is associated with increased risk of both mental and somatic disorders, such as depression, anxiety, obesity, diabetes, cardiovascular disorders, and cancer [1-6]. Whether insufficient sleep increases the risk of infections has received less attention. An extensive review on how sleep and the immune system interact calls for more studies [7]. Based on existing scientific evidence, insufficient sleep duration and/or quality are assumed to increase the risk of infections [7-10]. We recently published data from two cross-sectional studies among adults showing that insomnia symptoms, short (<6 h) and long (>9 h) sleep durations are associated with increased risk of several types of infections, in comparison to no insomnia symptoms and a sleep duration of 6–9 h, respectively [11, 12]. Among American adults, short (5 h or less) and long (9 h or more) sleep durations are associated with increased odds of reporting a cold [13]. Of note, sleep duration may differ significantly from sleep need [14]. Thus, sleep debt, calculated as the difference between self-reported sleep need and sleep duration, may better estimate risks of infections compared with sleep duration *per se*. In line with this notion, we showed strong associations between increasing sleep debt and infections in the cross-sectional study. Sleep debt of more than 2 h was associated with increased odds of common cold, throat infection, ear infection, sinusitis, pneumonia/bronchitis, influenza-like illness, skin infection, and gastrointestinal infection [12].

A possible causal relationship between poor sleep and infections is evidenced from two large population cohorts, in which registry-based diagnosis of insomnia predisposed for influenza, upper respiratory infections and severe COVID-19 [15]. A study with close to 400 000 participants from the UK Biobank showed that healthy sleep patterns reduced the risk of hospitalization for infections [16]. The study highlighted the potential of sleep interventions in preventing infectious diseases. Data from the Nurses' Health Study II cohort showed that habitual short (5 h or less) and long (9 h or more) sleep durations were both associated with increased risk of pneumonia diagnosed by chest X-rays [17]. In addition, increased pneumonia risk was present among participants who responded that their sleep duration was not adequate. Furthermore, studies on influenza and hepatitis vaccinations indicate that short sleep duration around the time of vaccination reduces the antibody response [7, 18]. These studies emphasize the possible association between sleep and infections. However, more studies in the general population are warranted, especially longitudinal studies, to investigate the link between insufficient sleep and common types of infections.

The aims of the present study were therefore to investigate whether sleep duration (short, normal, long), sleep debt (no, moderate, large), and insomnia symptoms (no, subthreshold, moderate to severe) at baseline would predict a wide variety of infections at follow-up 1 year later. Based on existing research, we hypothesized that both short and long sleep durations (<6 h and >9 h), but even more so increasing sleep debt, would increase the risk of infections. Furthermore, we hypothesized that increasing insomnia severity would predict increased risk of infections.

## Materials and methods

### Study design and participants

The Norwegian Primary Care Research Network—PraksisNett (www.praksisnett.no) facilitates recruitment of primary care patients to research [19]. In the present study, PraksisNett was used to identify potential participants aged between 25 and 70 years to a study focusing on sleep and infections. A total of 29 GPs were provided lists of eligible patients electronically, and the GPs subsequently sent invitations to patients through the National online health services (www.helsenorge.no) in Norway with a link to the online survey. Only the GPs knew the identity of the identified patients until consent was given. The GPs had the opportunity to exclude patients who they considered not eligible for participation (e.g. due to terminal disease, acute life crisis, etc.). The GPs were instructed to invite 60 patients each, with 20 from a list of patients who had been diagnosed with a sleep problem during the last year (code P06 in the International Classification of Primary Care, 2nd edition—ICPC-2) and 40 from a list of patients without a diagnosed sleep problem during the last year. We used this procedure to ensure enough patients with sleep problems. Twelve of the GPs invited patients on two separate occasions. The initial invitations were sent out between March 2022 and January 2023. Patients who entered the online survey (provided by SurveyXact by Ramboll; https://rambollxact.no) were first presented with information about the study, and only patients who consented to participate received the survey questions. Baseline data from this study are already published [12]. In total, 861 patients completed the baseline survey and agreed to be contacted 1 year later. These patients were therefore invited (via email through SurveyXact) to a follow-up survey 1 year after completing the baseline survey. In case of no response, one reminder email was sent. The response rate at follow-up was 60.5%, rendering 521 patients eligible for the present study.

### Survey items analyzed in the present study

At baseline, the participants were asked about gender (male; female; do not want to say/other), age (25–70 years) and if they had children living at home (no; yes). Habitual daily sleep duration and sleep need (how many hours of sleep per day do you need to feel rested?) were self-reported from drop-down menus with 15 min intervals (“0 min” up until “more than 12 h”) and categorized into <6 h, 6–9 h, and >9 h. Sleep debt was subsequently calculated as the difference between sleep need and sleep duration and split into three categories (no sleep debt; ≤2 h sleep debt; >2 h sleep debt).

Insomnia Severity Index (ISI) is a seven-item instrument which assesses perceived severity of nocturnal and daytime symptoms of insomnia during the last two weeks [20]. Each item is rated on a scale from 0 to 4 with a total score ranging from 0 to 28. A score of 0–7 indicates no insomnia, 8–14 subthreshold insomnia, and 15–28 indicates moderate to severe insomnia symptoms. Cronbach's alpha for the ISI was 0.91 in the present sample.

At follow-up, the participants responded to whether they had experienced the following infections during the last 3 months (no; yes): common cold, throat infection, ear infection, sinusitis, pneumonia/bronchitis, COVID-19, influenza-like illness, skin infection (erysipelas, herpes labialis, etc.), gastrointestinal infection with vomiting and/or diarrhea, urinary infection (cystitis, pyelonephritis), venereal disease (chlamydia, genital herpes), and eye infection. Thereafter, we calculated the proportion of participants who had experienced at least one of these infections (no; yes) during the last 3 months. Furthermore, the participants were asked whether they experience more infections than other people at the same age (more infections than peers) with four response alternatives (no; a bit more often; somewhat more often; much more often). This variable was categorized as “no” and “yes” (the other three response alternatives).

### Ethics

The study was approved by the Regional Committee for Medical and Health Research Ethics (REK sør-øst, application number 268606). All participants consented before receiving the survey questions.

### Statistics

Data analyses were conducted with SPSS, version 29 (IBM SPSS Statistics). The associations between sleep duration (<6 h vs. 6–9 h vs. >9 h) and different types of infections (only infections reported by at least 50 participants) were explored using Pearson's *χ*² test. Similarly, the associations between sleep debt (no sleep debt vs. ≤2 h vs. >2 h) and different types of infections, and associations between insomnia severity (ISI: no vs. subthreshold vs. moderate to severe insomnia symptoms) and different types of infections were explored with Pearson's *χ*² test. Lastly, crude and adjusted (for gender, age, and children living at home) logistic regression analyses were conducted with the different infections at follow-up as dependent variables (no = 0; yes = 1) and sleep duration (6–9 h as reference), sleep debt (no sleep debt as reference), and insomnia severity (no insomnia as reference) at baseline as independent variables. The significance level was set to 0.05 (two-sided).

## Results

Table 1 presents the characteristics of the study sample at baseline. Mean age was 49.9 (SD = 11.7) years. Short sleep duration (<6 h) was reported by 21.5% and long sleep duration (>9 h) by 2.5%. Based on sleep need and sleep duration, sleep debts of ≤2 h and >2 h were calculated to 56.4% and 12.7%, respectively. According to ISI, 44.0% of the patients had no insomnia, 37.2% had subthreshold insomnia, and 18.8% had moderate to severe insomnia symptoms.

**Table 1:** Characteristics of the survey participants invited by the general practitioner at baseline (*n* = 521).

	Number	Percentage (%)
**Gender**		
Male	213	40.9
Female	308	59.1
Do not want to say/other	0	0.0
**Age (years)**		
25–40	120	23.0
41–55	217	41.7
56–70	184	35.3
**Children living at home**		
No	320	61.4
Yes	201	38.6
**Sleep duration**		
<6 h	112	21.5
6–9 h	396	76.0
>9 h	13	2.5
**Sleep need**		
<6 h	12	2.3
6–9 h	484	92.9
>9 h	25	4.8
**Sleep debt**		
No sleep debt	161	30.9
≤2 h	294	56.4
>2 h	66	12.7
**Insomnia Severity Index**		
No insomnia	229	44.0
Subthreshold insomnia	194	37.2
Moderate to severe Insomnia	98	18.8

Table 2 presents the percentage of self-reported infections experienced during the last 3 months at 1-year follow-up, ranging from 1.6% for venereal disease to 44.4% for common cold. A total of 68.9% reported to have had one or more of these self-reported infections. In total, 23.3% of the participants reported that they experience more infections than peers.

**Table 2:** Self-reported infections (*n* = 489) during the last 3 months at 1-year follow-up.

	Number	Percentage (%)
**Any type of infection**		
No	152	31.1
Yes	337	68.9
**Common cold**		
No	272	55.6
Yes	217	44.4
**Throat infection**		
No	441	90.2
Yes	48	9.8
**Ear infection**		
No	469	95.9
Yes	20	4.1
**Sinusitis**		
No	454	92.8
Yes	35	7.2
**Pneumonia/bronchitis**		
No	480	98.2
Yes	9	1.8
**COVID-19**		
No	441	90.2
Yes	48	9.8
**Influenza-like illness**		
No	342	69.9
Yes	147	30.1
**Skin infection**		
No	390	79.8
Yes	99	20.2
**Gastrointestinal infection**		
No	404	82.6
Yes	85	17.4
**Urinary infection**		
No	467	95.5
Yes	22	4.5
**Venereal disease**		
No	481	98.4
Yes	8	1.6
**Eye infection**		
No	464	94.9
Yes	25	5.1
**More infections than peers (41 missing)**		
No	368	76.7
Yes	112	23.3

Table 3 presents sleep duration categories at baseline and the prevalence of different types of self-reported infections (only infections reported by at least 50 participants are presented) at 1-year follow-up. Significant differences with sleep duration were present for influenza-like illness, skin infection, gastrointestinal infection, and for experiencing more infections than peers. For influenza-like illness, gastrointestinal infection and experiencing more infections than peers, participants with long sleep duration reported the highest prevalence. For skin infection, participants with long sleep duration reported the lowest prevalence.

**Table 3:** Self-reported sleep duration at baseline and self-reported infections 1 year later (*n* = 489).

	Sleep duration				
	<6 h (*n*=104)	6–9 h (*n*=374)	>9 h (*n*=11)	χ^2^ (df)	*P*-value
**Any type of infection**				3.4 (2)	.183
No	29 (27.9%)	122 (32.6%)	1 (9.1%)		
Yes	75 (72.1%)	252 (67.4%)	10 (90.9%)		
**Common cold**				0.5 (2)	.767
No	57 (54.8%)	210 (56.1%)	5 (45.5%)		
Yes	47 (45.2%)	164 (43.9%)	6 (54.5%)		
**Influenza-like illness**				**6.1 (2)**	**.049**
No	73 (70.2%)	265 (70.9%)	4 (36.4%)		
Yes	31 (29.8%)	109 (29.1%)	7 (63.6%)		
**Skin infection**				**9.5 (2)**	**.008**
No	72 (69.2%)	308 (82.4%)	10 (90.9%)		
Yes	32 (30.8%)	66 (17.6%)	1 (9.1%)		
**Gastrointestinal infection**				**7.3 (2)**	**.026**
No	83 (79.8%)	315 (84.2%)	6 (54.5%)		
Yes	21 (20.2%)	59 (15.8%)	5 (45.5%)		
**More infections than peers**				**7.5 (2)**	**.023**
No	73 (73.0%)	290 (78.6%)	5 (45.5%)		
Yes	27 (27.0%)	79 (21.4%)	6 (54.5%)		

Only self-reported infections with at least 50 participants are reported. χ^2^, Pearson’s chi-square. df, degrees of freedom. Significant results are indicated in bold.

Table 4 presents the prevalence of self-reported infections at 1-year follow-up stratified according to sleep debt at baseline. A pattern of lower prevalence of infection was observed among participants with no sleep debt, but significant findings were present only for skin infection and experiencing more infections than peers.

**Table 4:** Self-reported sleep debt at baseline and self-reported infections 1 year later (*n* = 489).

	Sleep debt				
	No (*n*=148)	≤2 h (*n*=282)	>2 h (*n*=59)	χ^2^ (df)	*P*-value
**Any type of infection**				2.6 (2)	.266
No	53 (35.8%)	84 (29.8%)	15 (25.4%)		
Yes	95 (64.2%)	198 (70.2%)	44 (74.6%)		
**Common cold**				4.5 (2)	.107
No	93 (62.8%)	148 (52.5%)	31 (52.5%)		
Yes	55 (37.2%)	134 (47.5%)	28 (47.5%)		
**Influenza-like illness**				2.9 (2)	.234
No	110 (74.3%)	195 (69.1%)	37 (62.7%)		
Yes	38 (25.7%)	87 (30.9%)	22 (37.3%)		
**Skin infection**				**9.1 (2)**	**.011**
No	129 (87.2%)	212 (75.2%)	49 (83.1%)		
Yes	19 (12.8%)	70 (24.8%)	10 (16.9%)		
**Gastrointestinal infection**				4.3 (2)	.116
No	130 (87.8%)	228 (80.9%)	46 (78.0%)		
Yes	18 (12.2%)	54 (19.1%)	13 (22.0%)		
**More infections than peers**				**11.5 (2)**	**.003**
No	123 (83.7%)	210 (76.1%)	35 (61.4%)		
Yes	24 (16.3%)	66 (23.9%)	22 (38.6%)		

Only self-reported infections with at least 50 participants are reported. χ^2^, Pearson’s chi-square. df, degrees of freedom. Significant results are indicated in bold.

Table 5 presents the prevalence of self-reported infections at 1-year follow-up stratified according to insomnia severity at baseline. Prevalence of influenza-like illness, gastrointestinal infection, and experiencing more infections than peers were dose-dependently increased with insomnia severity.

**Table 5:** Self-reported insomnia severity at baseline and self-reported infections 1 year later (*n* = 489).

	Insomnia Severity Index				
	No (*n*=216)	Subthreshold (*n*=182)	Insomnia (*n*=91)	χ^2^ (df)	*P*-value
**Any type of infection**				5.0 (2)	.082
No	77 (35.6%)	54 (29.7%)	21 (23.1%)		
Yes	139 (64.4%)	128 (70.3%)	70 (76.9%)		
**Common cold**				0.4 (2)	.826
No	122 (56.5%)	102 (56.0%)	48 (52.7%)		
Yes	94 (43.5%)	80 (44.0%)	43 (47.3%)		
**Influenza-like illness**				**6.3 (2)**	**.042**
No	159 (73.6%)	129 (70.9%)	54 (59.3%)		
Yes	57 (26.4%)	53 (29.1%)	37 (40.7%)		
**Skin infection**				3.5 (2)	.176
No	180 (83.3%)	142 (78.0%)	68 (74.7%)		
Yes	36 (16.7%)	40 (22.0%)	23 (25.3%)		
**Gastrointestinal infection**				**15.8 (2)**	**<.001**
No	194 (89.8%)	144 (79.1%)	66 (72.5%)		
Yes	22 (10.2%)	38 (20.9%)	25 (27.5%)		
**More infections than peers**				**16.7 (2)**	**<.001**
No	179 (84.0%)	134 (74.9%)	55 (62.5%)		
Yes	34 (16.0%)	45 (25.1%)	33 (37.5%)		

Only self-reported infections with at least 50 participants are reported. χ^2^, Pearson’s chi-square. df, degrees of freedom. Significant results are indicated in bold.

Table 6 presents logistic regression analyses with the different types of self-reported infections at 1-year follow-up as the dependent variables. In the adjusted analyses with 6–9 h of sleep duration at baseline as reference, long sleep duration increased the odds of influenza-like illness, gastrointestinal infection, and experiencing more infections than peers by about four times, whereas short sleep duration doubled the odds of skin infection. Compared to no sleep debt at baseline, ≤2 h of sleep debt doubled the odds of skin infection and >2 h of sleep debt tripled the odds of experiencing more infections than peers. Lastly, moderate to severe insomnia symptoms at baseline doubled the odds of any type of infection and influenza-like illness, and more than tripled the odds of gastrointestinal infection and experiencing more infections than peers. Subthreshold insomnia increased odds of gastrointestinal infection and experiencing more infections than peers (Table 6).

**Table 6:** Logistic regression analyses with self-reported infection (no = 0; yes = 1) at 1-year follow-up as dependent variable and sleep duration, sleep debt, and insomnia severity at baseline as predictors (*n* = 489).

	Any type of infection (*n* = 337) OR (95% CI)		Common cold (*n* = 217) OR (95% CI)		Influenza-like illness (*n* = 147) OR (95% CI)	
	Crude	Adjusted	Crude	Adjusted	Crude	Adjusted
**Sleep duration**						
6–9 h	1.00 (ref)	1.00 (ref)	1.00 (ref)	1.00 (ref)	1.00 (ref)	1.00 (ref)
<6 h	1.25 (0.78–2.02)	1.32 (0.81–2.16)	1.06 (0.68–1.63)	1.14 (0.73–1.79)	1.03 (0.64–1.66)	1.11 (0.69–1.81)
>9 h	4.84 (0.61–38.25)	4.78 (0.60–38.37)	1.54 (0.46–5.12)	1.55 (0.45–5.28)	**4.26 (1.22–14.83)**	**3.99 (1.13–14.11)**
**Sleep debt**						
No sleep debt	1.00 (ref)	1.00 (ref)	1.00 (ref)	1.00 (ref)	1.00 (ref)	1.00 (ref)
≤2 h	1.32 (0.86–2.01)	1.22 (0.79–1.88)	**1.53 (1.02–2.30)**	1.42 (0.94–2.15)	1.29 (0.83–2.02)	1.22 (0.77–1.93)
>2 h	1.64 (0.83–3.22)	1.51 (0.76–3.01)	1.53 (0.83–2.81)	1.48 (0.80–2.77)	1.72 (0.90–3.28)	1.68 (0.87–3.23)
**Insomnia severity**						
No insomnia	1.00 (ref)	1.00 (ref)	1.00 (ref)	1.00 (ref)	1.00 (ref)	1.00 (ref)
Subthreshold	1.31 (0.86–2.00)	1.31 (0.85–2.02)	1.02 (0.68–1.52)	1.05 (0.70–1.58)	1.15 (0.74–1.78)	1.15 (0.74–1.80)
Insomnia	**1.85 (1.05–3.24)**	**1.95 (1.10–3.45)**	1.16 (0.71–1.90)	1.26 (0.76–2.08)	**1.91 (1.14–3.20)**	**1.96 (1.16–3.33)**

	Skin infection (*n* = 99) OR (95% CI)		Gastrointestinal infection (*n* = 85) OR (95% CI)		More infections than peers (*n* = 112) OR (95% CI)	
	Crude	Adjusted	Crude	Adjusted	Crude	Adjusted
**Sleep duration**						
6–9 h	1.00 (ref)	1.00 (ref)	1.00 (ref)	1.00 (ref)	1.00 (ref)	1.00 (ref)
<6 h	**2.07 (1.27–3.40)**	**2.03 (1.22–3.38)**	1.35 (0.78–2.35)	1.39 (0.79–2.44)	1.36 (0.82–2.25)	1.46 (0.87–2.45)
>9 h	0.47 (0.06–3.71)	0.38 (0.05–3.10)	**4.45 (1.32–15.05)**	**4.31 (1.25–14.86)**	**4.41 (1.31–14.81)**	**3.74 (1.09–12.82)**
**Sleep debt**						
No sleep debt	1.00 (ref)	1.00 (ref)	1.00 (ref)	1.00 (ref)	1.00 (ref)	1.00 (ref)
≤2 h	**2.24 (1.29–3.89)**	**2.13 (1.21–3.74)**	1.71 (0.96–3.04)	1.56 (0.87–2.81)	1.61 (0.96–2.70)	1.50 (0.88–2.54)
>2 h	1.39 (0.60–3.19)	1.17 (0.50–2.73)	2.04 (0.93–4.49)	1.84 (0.83–4.09)	**3.22 (1.62–6.42)**	**2.95 (1.46–5.96)**
**Insomnia severity**						
No insomnia	1.00 (ref)	1.00 (ref)	1.00 (ref)	1.00 (ref)	1.00 (ref)	1.00 (ref)
Subthreshold	1.41 (0.85–2.33)	1.30 (0.78–2.17)	**2.33 (1.32–4.11)**	**2.35 (1.32–4.19)**	**1.77 (1.07–2.91)**	**1.68 (1.01–2.79)**
Insomnia	1.69 (0.94–3.06)	1.55 (0.84–2.84)	**3.34 (1.77–6.32)**	**3.54 (1.84–6.79)**	**3.16 (1.79–5.57)**	**3.09 (1.73–5.51)**

Only self-reported infections with at least 50 participants are reported. OR, odds ratio. CI, confidence interval.

The logistic regression analyses were adjusted for gender, age category, and children living at home. Significant results are indicated in bold.

## Discussion

Our hypotheses were partly confirmed—as participants with normal sleep duration, no sleep debt, and no insomnia symptoms at baseline reported lower prevalences of most infections at 1-year follow-up compared to participants with short or long sleep duration, sleep debt, and insomnia symptoms, respectively. However, statistically significant findings were limited to a few of the self-reported infections. Importantly, the adjusted analyses suggested that insomnia severity dose-dependently predicted increased prevalence of any type of infection, influenza-like illness, gastrointestinal infection with vomiting and/or diarrhea, and experiencing more infections than peers. Similarly, increasing sleep debt dose-dependently predicted experiencing more infections than peers.

Findings in the present study are in line with other studies suggesting an association between sleep and risk of infection [11-13]. Sleep is recognized as essential for immune function [1, 7], but longitudinal studies investigating whether insufficient sleep predicts infections are few. Some population-based cohort studies have indicated causality between poor sleep and infections. Data from the cohorts UK Biobank and FinnGen showed that a registry-based insomnia diagnosis predicted influenza, upper respiratory infections, and severe COVID-19 [15]. Furthermore, another study from the UK Biobank showed that poor sleep patterns increased the risk of hospital admissions due to infections [16]. Self-reported short and long sleep durations have been shown to increase the risk of incident pneumonia over the next 4 years [17]. In addition, an experimental study showed that people sleeping less than 6 h have increased susceptibility to rhinovirus infection [21].

The present study consists of fewer participants than the above-mentioned cohorts but adds to the knowledge base of causality between insufficient sleep and risk of infections. One strength with our paper was that we included many different types of commonly experienced infections. We also added a question about experiencing more infections than peers, which we believe may capture a vulnerability for infections that some people claim they have. Such a vulnerability may possibly occur due to impaired immune function. In our study, long sleep duration, sleep debt, and insomnia severity at baseline all strongly predicted subjective experience of more infections than peers at follow-up.

Our previous cross-sectional study showed that increasing sleep debt was dose-dependently associated with the prevalence of several types of infections [12]. This was not as evident in the present longitudinal study, however, participants with no sleep debt reported the lowest prevalence of all assessed infections. No other longitudinal study has investigated sleep debt and infection risk. Some individuals may need less sleep than others, e.g. short sleepers, meaning that they regularly feel alert and refreshed after sleeping less than 6 h. Therefore, focusing solely on sleep duration may not capture insufficient sleep [14]. We believe that sleep is associated with increased infection risk, especially among those individuals who get less sleep than they need [12]. Other studies also suggest that increasing levels of sleep debt are associated with poorer health outcomes [22, 23].

Even though some of the associations between insufficient sleep and infections were not statistically significant, we believe this may be due to the low number of participants. For instance, for any type of infection, the prevalences were 64.2%, 70.2%, and 74.6%, for no sleep debt, ≤2 h, and >2 h of sleep debt, respectively. We think these numbers may suggest clinically important differences, although they did not reach statistical significance.

Somewhat surprisingly, insomnia severity dose-dependently predicted gastrointestinal infection with vomiting and/or diarrhea. Sleep disturbances are quite common in patients with gastrointestinal disease [24]. The gut-brain axis has received increased attention in recent years, but most studies focus on whether regulating the intestinal flora can impact sleep [25, 26]. Not many studies have investigated whether sleep problems increase the risk of gastrointestinal infections. Our data suggest a possible cause-and-effect, but more studies are needed to elucidate the associations between sleep and gastrointestinal infections.

Long sleep duration (>9 h) predicted increased odds of influenza-like illness, gastrointestinal infection, and experiencing more infections than peers. However, long sleep duration is more common among patients with different diseases [27] and is also associated with depression, low educational level, low physical activity level, and high drinking and smoking rates [28]. We were not able to adjust for such factors, which likely would have impacted our findings. Furthermore, due to the low number of participants reporting long sleep duration, we recommend caution in interpreting the association with infections.

This study has several strengths and limitations. The longitudinal design is an obvious strength. Another strength was that we assessed insomnia with a validated instrument [20]. However, the ISI may not accurately differentiate between insomnia and other sleep disorders with insomnia symptoms, such as obstructive sleep apnea or circadian rhythm sleep-wake disorders [29]. Although approximately 40% of participants did not complete the follow-up survey, the resulting response rate of 60.5% is in line with or above typical rates for longitudinal survey studies [30], though some risk of selection bias cannot be excluded. Still, our study was likely underpowered with only about 500 participants. Many of the infections were reported by less than 50 participants, and we chose not to analyze associations with insufficient sleep for those infections. Another limitation was that we were unable to adjust for baseline medical conditions or other comorbidities, as such data were not collected. Residual confounding due to underlying health status cannot therefore be ruled out. All data were based on self-reports, which represents a major limitation. Neither sleep duration nor infections were objectively assessed. While many public health recommendations encourage adults to obtain at least 7 h of sleep, a large body of sleep and health research shows that the strongest and most consistent increases in adverse health outcomes occur when individuals report <6 h of sleep [31, 32]. Therefore, short sleep duration was defined as <6 h of sleep. Moreover, as several outcomes were common, logistic regression may overestimate relative risks, and the odds ratios should therefore be interpreted with caution [33]. Other limitations include recall bias [34] and social desirability bias [35]. Our objective was to evaluate whether baseline sleep characteristics predict infection risk over the following year. Future studies should include larger samples and repeated sleep assessments to explore sleep trajectories, to study how possible changes in sleep debt and/or insomnia symptoms over time influence infection risk.

In conclusion, we found that both short and long sleep duration, sleep debt, and insomnia severity predicted increased prevalence of infections 1 year later. For influenza-like illness, gastrointestinal infection with vomiting and/or diarrhea, and experiencing more infections than peers, the prevalences increased with insomnia severity. Furthermore, increasing sleep debt dose-dependently predicted experiencing more infections than peers. These findings underline the notion that insufficient sleep is a risk factor for future infections.

## Data Availability

The data underlying this article will be shared at reasonable request to the corresponding author.

## References

[1] Irwin MR, Opp MR. Sleep health: reciprocal regulation of sleep and innate immunity. Neuropsychopharmacology 2017;42:129–55. 10.1038/npp.2016.148 27510422 PMC5143488

[2] Li M, Zhang XW, Hou WS et al Insomnia and risk of cardiovascular disease: a meta-analysis of cohort studies. Int J Cardiol 2014;176:1044–7. 10.1016/j.ijcard.2014.07.284 25156850

[3] Riemann D, Baglioni C, Bassetti C et al European guideline for the diagnosis and treatment of insomnia. J Sleep Res 2017;26:675–700. 10.1111/jsr.12594 28875581

[4] Anothaisintawee T, Reutrakul S, Van Cauter E et al Sleep disturbances compared to traditional risk factors for diabetes development: systematic review and meta-analysis. Sleep Med Rev 2016;30:11–24. 10.1016/j.smrv.2015.10.002 26687279

[5] Wu Y, Zhai L, Zhang D. Sleep duration and obesity among adults: a meta-analysis of prospective studies. Sleep Med 2014;15:1456–62. 10.1016/j.sleep.2014.07.018 25450058

[6] Wu TT, Zou YL, Xu KD et al Insomnia and multiple health outcomes: umbrella review of meta-analyses of prospective cohort studies. Public Health 2023;215:66–74. 10.1016/j.puhe.2022.11.021 36645961

[7] Besedovsky L, Lange T, Haack M. The sleep-immune crosstalk in health and disease. Physiol Rev 2019;99:1325–80. 10.1152/physrev.00010.2018 30920354 PMC6689741

[8] Salehinejad MA, Azarkolah A, Ghanavati E et al Circadian disturbances, sleep difficulties and the COVID-19 pandemic. Sleep Med 2022;91:246–52. 10.1016/j.sleep.2021.07.011 34334305 PMC8277544

[9] Robinson CH, Albury C, McCartney D et al The relationship between duration and quality of sleep and upper respiratory tract infections: a systematic review. Fam Pract 2021;38:802–10. 10.1093/fampra/cmab033 33997896 PMC8656143

[10] Lee RU, Glickman GL. Sleep, circadian health and melatonin for mitigating COVID-19 and optimizing vaccine efficacy. Front Neurosci 2021;15:711605. 10.3389/fnins.2021.711605 34489630 PMC8416504

[11] Forthun I, Eliassen KER, Emberland KE et al The association between self-reported sleep problems, infection, and antibiotic use in patients in general practice. Front Psychiatry 2023;14:1033034. 10.3389/fpsyt.2023.1033034 36937728 PMC10017838

[12] Bjorvatn B, Rortveit G, Rebnord I et al Self-reported short and long sleep duration, sleep debt and insomnia are associated with several types of infections: results from the Norwegian practice-based research network in general practice - PraksisNett. Sleep Med X 2023;5:100074. 10.1016/j.sleepx.2023.100074 37223609 PMC10200965

[13] Prather AA, Carroll JE. Associations between sleep duration, shift work, and infectious illness in the United States: data from the National Health Interview Survey. Sleep Health 2021;7:638–43. 10.1016/j.sleh.2021.05.004 34193397

[14] Ursin R, Bjorvatn B, Holsten F. Sleep duration, subjective sleep need, and sleep habits of 40- to 45-year-olds in the Hordaland Health Study. Sleep 2005;28:1260–9. 10.1093/sleep/28.10.1260 16295211

[15] Jones SE, Maisha FI, Strausz SJ, et al The public health impact of poor sleep on severe COVID-19, influenza and upper respiratory infections. EBioMedicine 2023;93:104630. 10.1016/j.ebiom.2023.104630

[16] Li HM, Zhang XR, Liao DQ et al Healthy sleep patterns and risk of hospitalization for infection: a large community-based cohort study. Transl Psychiatry 2025;15:100. The UK Biobank received ethical approval from the research ethics committee (REC reference for UK Biobank 11/NW/0382), and the participants provided written informed consent. Our study adhered to the STROBE guidelines for the cohort studies [54]. 10.1038/s41398-025-03314-6 40148289 PMC11950331

[17] Patel SR, Malhotra A, Gao X et al A prospective study of sleep duration and pneumonia risk in women. Sleep 2012;35:97–101. 10.5665/sleep.1594 22215923 PMC3242694

[18] Spiegel K, Sheridan JF, Van Cauter E. Effect of sleep deprivation on response to immunization. JAMA 2002;288:1471–2. 10.1001/jama.288.12.1471-a 12243633

[19] Kristoffersen ES, Bjorvatn B, Halvorsen PA et al The Norwegian PraksisNett: a nationwide practice-based research network with a novel IT infrastructure. Scand J Prim Health Care 2022;40:217–26. 10.1080/02813432.2022.2073966 35549798 PMC9397441

[20] Morin CM, Belleville G, Belanger L et al The insomnia severity Index: psychometric indicators to detect insomnia cases and evaluate treatment response. Sleep 2011;34:601–8. 10.1093/sleep/34.5.601 21532953 PMC3079939

[21] Prather AA, Janicki-Deverts D, Hall MH et al Behaviorally assessed sleep and susceptibility to the common cold. Sleep 2015;38:1353–9. 10.5665/sleep.4968 26118561 PMC4531403

[22] Hublin C, Kaprio J, Partinen M et al Insufficient sleep–a population-based study in adults. Sleep 2001;24:392–400. 10.1093/sleep/24.4.392 11403523

[23] van Leeuwen WM, Lehto M, Karisola P et al Sleep restriction increases the risk of developing cardiovascular diseases by augmenting proinflammatory responses through IL-17 and CRP. PLoS One 2009;4:e4589. 10.1371/journal.pone.0004589 19240794 PMC2643002

[24] Khanijow V, Prakash P, Emsellem HA et al Sleep dysfunction and gastrointestinal diseases. Gastroenterol Hepatol (N Y) 2015;11:817–25. 27134599 PMC4849511

[25] Yu B, Wang KY, Wang NR et al Effect of probiotics and paraprobiotics on patients with sleep disorders and sub-healthy sleep conditions: a meta-analysis of randomized controlled trials. Front Neurol 2024;15:1477533. 10.3389/fneur.2024.1477533 39479010 PMC11521871

[26] Irwin C, McCartney D, Desbrow B et al Effects of probiotics and paraprobiotics on subjective and objective sleep metrics: a systematic review and meta-analysis. Eur J Clin Nutr 2020;74:1536–49. 10.1038/s41430-020-0656-x 32433598

[27] Jike M, Itani O, Watanabe N et al Long sleep duration and health outcomes: a systematic review, meta-analysis and meta-regression. Sleep Med Rev 2018;39:25–36. 10.1016/j.smrv.2017.06.011 28890167

[28] Liu TZ, Xu C, Rota M et al Sleep duration and risk of all-cause mortality: a flexible, non-linear, meta-regression of 40 prospective cohort studies. Sleep Med Rev 2017;32:28–36. 10.1016/j.smrv.2016.02.005 27067616

[29] Bjorvatn B, Jernelov S, Pallesen S. Insomnia - A heterogenic disorder often comorbid with psychological and somatic disorders and diseases: a narrative review with focus on diagnostic and treatment challenges. Front Psychol 2021;12:639198. 10.3389/fpsyg.2021.639198 33643170 PMC7904898

[30] Baruch Y . Response rate in academic studies-A comparative analysis. Hum Relat 1999;52:421–38. 10.1177/001872679905200401

[31] Yang L, Xi B, Zhao M et al (January 13, 2021) Association of sleep duration with all-cause and disease-specific mortality in US adults. J Epidemiol Community Health, 10.1136/jech-2020-215314 33441393

[32] Qasrawi SO, BaHammam AS. Role of sleep and sleep disorders in cardiometabolic risk: a review and update. Curr Sleep Med Rep 2024;10:34–50. 10.1007/s40675-024-00276-x

[33] Davies HT, Crombie IK, Tavakoli M. When can odds ratios mislead? BMJ 1998;316:989–91. 10.1136/bmj.316.7136.989 9550961 PMC1112884

[34] Talari K, Goyal M. Retrospective studies - utility and caveats. J R Coll Physicians Edinb 2020;50:398–402. 10.4997/jrcpe.2020.409 33469615

[35] Krumpal I . Determinants of social desirability bias in sensitive surveys: a literature review. Qual Quant 2013;47:2025–47. 10.1007/s11135-011-9640-9

